# 581 Sustained Multidisciplinary CLABSI Bundle in the Burn Center

**DOI:** 10.1093/jbcr/irac012.209

**Published:** 2022-03-23

**Authors:** Sylvia H Dao, Jamie M Heffernan, Andrew Greenway, Kaitlyn M Libraro, Deirdre Twohig, Jim Gallagher

**Affiliations:** New York Presbyterian, New York, New York; NY Presbyterian Weill Cornell, New York, New York; New York Presbyterian Hospital, New York, New York; New York Presbyterian Hospital, New York, New York; NYP Weill Cornell Medical Center, New York, New York; none, Phoenicia, New York

## Abstract

**Introduction:**

Central line-associated bloodstream infections (CLABSI) remains a high risk for burn patients due to their compromised integumentary system. Additionally, identifying bloodstream infections secondary to a burn infection versus a central line remains a challenge. Literature review suggests multidisciplinary CLABSI bundles (MCB) demonstrate reduction of CLABSIs. This research looks at the use of a MCB compared to the application of the standard CLABSI bundle alone reduce CLABSI incidence and standardized infection ratios over the past two years in the Burn Center. We hypothesize a MCB will have sustained CLABSI SIR and incidence reduction over time.

**Methods:**

Infection Prevention & Control CLABSI data collection on all burn patients pre-intervention (2017-2018) and post-intervention (2019-2020) were performed as a quality improvement project; IRB was not obtained. In addition to the CLABSI Bundle (optimal site, aseptic insertion, aseptic maintenance, timely removal, & education), MCB was also implemented in Qtr. 1, 2019. MCB includes necessity of line discussion during daily and multidisciplinary rounds, strategic blood culture collection upon admission/transfers for patients with existing central line, improved nursing and provider documentation of burn infection per CDC definitions, integration of CLABSI reviews in tiered Burn Quality Improvement Program, and universal zero CLABSI goal setting for RNs. Two-sample t-test will be used to compare results pre-intervention (2017-18) & post-intervention (2019-20). CLABSI incidence and standardized Infection ratio will be illustrated per year.

**Results:**

The post-intervention CLABSI SIR data (M= 0.08, SD=0.11) significantly improved when compared to the pre-intervention CLABSI SIR data (M= 0.77, SD= 0.169), t(2)=4.72, *p*=0.02. The post-intervention CLABSI incidence data (M=0.5, SD=0.70) significantly improved when compared to the pre-intervention CLABSI incidence data (M=4.5, SD= 0.70), t(2)= 5.65, *p*= 0.01

**Conclusions:**

The combination of the CLABSI bundle with the MCB demonstrated favorable CLABSI SIR and incidence reduction, as literature suggests. Implementing multimodal, multidisciplinary interventions must be a priority for sustained CLABSI reduction. Considerations for future studies should measure length of catheter days, incidence of positive blood cultures on admission/transfers for patients with existing central lines, and incidence of burn infection rates with positive blood culture and an existing central line.

